# *β*-Phenethyl Isothiocyanate Induces Cell Death in Human Osteosarcoma through Altering Iron Metabolism, Disturbing the Redox Balance, and Activating the MAPK Signaling Pathway

**DOI:** 10.1155/2020/5021983

**Published:** 2020-04-04

**Authors:** Huanhuan Lv, Chenxiao Zhen, Junyu Liu, Peng Shang

**Affiliations:** ^1^School of Life Sciences, Northwestern Polytechnical University, Xi'an 710072, China; ^2^Research & Development Institute of Northwestern Polytechnical University in Shenzhen, Shenzhen 518057, China; ^3^Research Center of Microfluidic Chip for Health Care and Environmental Monitoring, Yangtze River Delta Research Institute of Northwestern Polytechnical University in Taicang, Suzhou 215400, China; ^4^Key Laboratory for Space Bioscience and Biotechnology, Northwestern Polytechnical University, Xi'an 710072, China

## Abstract

Osteosarcoma is the most common primary malignancy of the skeleton in children and adults. The outcomes of people with osteosarcomas are unsatisfied. *β*-Phenethyl isothiocyanate (PEITC) exhibits chemoprevention and chemotherapeutic activities against many human cancers. The molecular mechanism underlying its action on osteosarcoma is still unknown. This study was aimed at investigating the effect of PEITC on human osteosarcoma both *in vitro* and *in vivo*. The results showed that PEITC reduced cell viability, inhibited proliferation, and caused G_2_/M cell cycle arrest in four human osteosarcoma cell lines (MNNG/HOS, U-2 OS, MG-63, and 143B). Then, we found that PEITC altered iron metabolism related to the processes of iron import, storage, and export, which resulted in increased labile iron. Expectedly, PEITC caused oxidative stress as a consequence of GSH depletion-inducing ROS generation and lipid peroxidation. Multiple cell death modalities, including ferroptosis, apoptosis, and autophagy, were triggered in human osteosarcoma cells. Three MAPKs (ERK, p38, and JNK) were all activated after PEITC treatment; however, they presented different responses among the four human osteosarcoma cell lines. ROS generation was proved to be the major cause of PEITC-induced decreased proliferative potential, altered iron metabolism, cell death, and activated MAPKs in human osteosarcoma cells. In addition, PEITC also significantly delayed tumor growth in a xenograft osteosarcoma mouse model with a 30 mg/kg administration dose. In conclusion, this study reveals that PEITC simultaneously triggers ferroptosis, apoptosis, and autophagy in human osteosarcoma cells by inducing oxidative stress.

## 1. Introduction

Osteosarcoma (OS) is a primary tumor of the skeleton ordinarily manifesting as malignant mesenchymal cells producing osteoid or immature bone [1]. Most of the cases range from 10 to 25 years of age. Although a large number of chemotherapeutic agents are in use, most of patients still need limb surgical resection and undergo lung metastasis [2]. Nowadays, there is no obvious improved outcome in OS patients [3, 4]. Alternative therapeutic approaches for OS patients are urgently needed to improve their life quality and increase their hope for future [5–7]. One of the promising explorations is to screen potential anticancer agents from natural products that can endow the marvelous power of plant phytochemicals to human health.

The daily diets of cruciferous vegetables profoundly benefit human health [8]. Epidemiological studies support the effects of long-term dietary intake of cruciferous vegetables in reducing the likelihood of cancer incidence, especially emphasizing the anticancer effect of their main active components isothiocyanates (ITCs) [9–12]. *β*-Phenethyl isothiocyanate (PEITC), one of the most promising ITCs, has been undergone small human clinical trials against cancers, such as breast cancer, lung cancer, and prostate cancer, either within cruciferous food or as a single agent [13, 14]. PEITC has its unique biological features charactering as high oral bioavailability, low clearance, and high protein binding [15]. Current evidence and outcomes suggest that it is potential for future clinical development and application in cancer therapy [16]. It has long been documented that the proposed antitumor mechanism of PEITC attributes to the modulation of cytochrome P450s, regulation of phase II detoxifying enzymes, induction of cell cycle arrest, and disturbance of redox homeostasis [17].

Although the biological effects of PEITC on cancers have been extensively demonstrated, there is limited knowledge concerning its effect on human OS. Herein, we intend to unravel the mechanism underlying the effects of PEITC on MNNG/HOS, U-2 OS, MG-63, and 143B human OS cells. Our results show that PEITC inhibits cell proliferation and triggers different cell death modalities including ferroptosis, apoptosis, and autophagy in four human OS cell lines. The cytotoxicity of PEITC on osteosarcoma cells attributes to GSH depletion, increase of labile iron, ROS generation, and MAPK signaling pathway activation. Understanding the antitumor mechanism of PEITC on human osteosarcoma cells is essential for the application of this natural compound for further clinical trials.

## 2. Materials and Methods

### 2.1. Cell Viability

MNNG/HOS, U-2 OS, MG-63, and 143B human OS cells were obtained from the cell bank of Chinese Academy of Sciences (Shanghai, China) and grown in Dulbecco's modified Eagle's medium (DMEM, Gibco, Grand Island, NY, USA) supplemented with 10% fetal bovine serum and 100 units/mL penicillin-streptomycin at 37°C in a 5% CO_2_ incubator. The viability of the four human OS cell lines after PEITC (Shanghai Hushi Laboratorial Equipment Co., Ltd, Shanghai, China) treatment was determined by using cell counting kit-8 (CCK-8 kit, Beyotime, C0037). OS cells were seeded in 96-well cell culture plates at a density of 5 × 10^4^ cells/mL and allowed to attach. The following day, cells were treated with different concentrations of PEITC for 24 h, 48 h, and 72 h. Then, 10% CCK-8 solution was added and incubated at 37°C for 2 h. Cell viability was determined by measuring absorbance at 450 nm on a Synergy HT multimode microplate reader (BioTek, Winooski, Vermont, USA).

### 2.2. Observation of Subcellular Structural Changes

MNNG/HOS OS cells were seeded in 6-well cell culture plates with a density of 1 × 10^5^ cells/mL and allowed to attach. The following day, cells were treated with 30 *μ*M PEITC for 4 h, 12 h, and 24 h, respectively. Cells were collected and washed by PBS, fixed in 2% paraformaldehyde, 2.5% glutaraldehyde in 0.1 M sodium cacodylate buffer before washing in 0.1 M sodium cacodylate buffer, then followed by postfixing in 1% osmium tetroxide in 0.1 M sodium cacodylate buffer. After fixation, cells were dehydrated through a graded series of alcohols and embedded in Spurrs Resin. Ultrathin sections were cut with a diamond knife using a Leica Ultracut S ultra-microtome and stained with both methanolic uranyl acetate and lead citrate before viewing in a LEO 906 transmission electron microscope operated at 60 kV.

### 2.3. Cell Death Assay

OS cells were seeded in 96-well cell culture plates with a density of 5 × 10^4^ cells/mL and allowed to attach. The following day, the cells were treated with 30 *μ*M PEITC in the presence of 50 *μ*M z-VAD-FMK (Beyotime, C1202), 50 *μ*M necrostatin-1 (Ner-1, SelleckChem, S8037), 10 *μ*M ferrostatin-1 (Fer-1, Sigma, SML0583), 100 nM liproxstatin-1 (Lip-1, SelleckChem, S7699), 1 *μ*M bafilomycin A1 (Baf-A1, SelleckChem, S1413), 10 mM 3-methyladenine (3-MA, SelleckChem, 2767), or 1 mM N-acetyl-L-cysteine (NAC, Sigma-Aldrich, A9165) for 24 h. Then, 10% CCK-8 solution was added and incubated at 37°C for 2 h. The cell culture plates were read by a Synergy HT multimode microplate reader (BioTek, Winooski, Vermont, USA) at a wavelength of 450 nm.

### 2.4. Colony Formation Assay

OS cells were plated at a density of 1 × 10^5^ cells/mL in 6-well cell culture plates and allowed to attach. The following day, the cells were treated with PEITC for 24 h. After treatment, the cells were detached using trypsin, and an equal number of cells from each treatment were reseeded in 6-well cell culture plates. The medium was changed every 3 days for consecutive 10 days. Finally, the colonies were stained with 0.5% crystal violet for 15 min, the dye was washed with pure water, images were photographed by a stereomicroscope (Canon Inc., Tokyo, Japan), and the colonies were counted.

### 2.5. EdU Cell Proliferation Assay

EdU cell proliferation assay was performed by using Cell-Light™ EdU ®Apollo567 *in vitro* imaging kit (RiboBio, C10310). In brief, OS cells were seeded at a density of 5 × 10^4^ cells/mL in 96-well cell culture plates and allowed to attach. The following day, the cells were treated with PEITC for 24 h. The cells were labeled with 50 *μ*M EdU solution for 2 h and then immobilized with PBS containing 4% paraformaldehyde for 30 min. Apollo staining solution was then added and incubated for 30 min protecting from light. In the final step, Hoechst 33342 solution was added, and cells were incubated in the dark for 30 min. Hoechst 33342 solution was removed, and the cells were washed three times with PBS. The images were captured immediately by an MD IL HC inverted fluorescence microscope (Leica, Wetzlar, Germany).

### 2.6. Cell Cycle Analysis

Cell cycle was analyzed by flow cytometer with PI/RNase staining buffer (Beyotime, C1052). In brief, OS cells were seeded at a density of 1 × 10^5^ cells/mL in 6-well cell culture plates and allowed to attach. The following day, the cells were treated with PEITC for 24 h. The cells were gently rinsed with precooled PBS solution, trypsinized, and centrifuged at 1,000 × g for 5 min to collect the cells. After washing once with cold PBS, the cells were resuspended in 1 mL of cold 70% ethanol and allowed to store at 4°C for 24 h. After washing the cells with cold PBS, 500 *μ*L of PI staining solution was added to each sample, and incubated for another 30 min at 37°C protecting from light. The samples were run on a FACSCalibur flow cytometer (BD Biosciences, San Jose, CA, USA), and the percentage of cells in each phase of cell cycle was calculated using the ModFit software.

### 2.7. Iron Quantification

The amount of total iron was determined by atomic absorption spectrometer (AAS, Analytik Jena, Germany). In brief, OS cells were seeded at a density of 1 × 10^5^ cells/mL in 6-well cell culture plates and allowed to attach. The following day, the cells were treated with PEITC for 24 h. After washing with PBS, the cells were resuspended in PBS for protein quantification and iron quantification. The samples for iron quantification were heat-disrupted with 500 *μ*L of HNO_3_ at 70°C for 2 h. The samples were analyzed by AAS, and total iron content was normalized to protein concentration.

### 2.8. Cellular Labile Iron Staining

Cellular labile iron was measured by Calcein-AM (Thermo, C3099) staining. In brief, OS cells were seeded at a density of 1 × 10^5^ cells/mL in 6-well cell culture plates, and after PEITC treatment, the cells were trypsinized, centrifuged, suspended in 1 mL of PBS, and counted. 1 × 10^6^ cells were collected and incubated with 100 nM Calcein-AM for 15 min at 37°C protected from light. After washing the extra Calcein-AM with PBS, the cells were centrifuged and resuspended in PBS again, and the fluorescence profile of the samples was monitored by using a FACSCalibur flow cytometer (BD Biosciences, San Jose, CA, USA).

### 2.9. Measurement of Cytosolic ROS

Intracellular cytosolic ROS was measured by using ROS assay kit (Beyotime, S0033). In brief, OS cells were seeded at a density of 1 × 10^5^ cells/mL in 6-well cell culture plates and allowed to attach. The following day, the cells were treated with PEITC for 24 h. After treatment, cells were collected, and DCFH-DA probe was loaded. The cells were incubated with DCFH-DA at 37°C for 20 min protected from light, then washed three times with serum-free medium to remove extracellular probes, and finally analyzed by a FACSCalibur flow cytometer (BD Biosciences, San Jose, CA, USA).

### 2.10. Measurement of Lipid ROS

Generation of lipid ROS was detected by using BODIPY 581/591 C11 sensor (Thermo Fisher, D3861). In brief, OS cells were seeded at a density of 1 × 10^5^ cells/mL in 6-well cell culture plates and allowed to attach. The following day, the cells were treated with PEITC for 24 h. The cells were then incubated with 10 *μ*M BODIPY 581/591 C11 staining solution for 40 min at 37°C protected from light. After staining, the cells were washed three times with PBS and immediately imaged by an MD IL HC inverted fluorescence microscope (Leica, Wetzlar, Germany).

### 2.11. Measurement of MDA

Malondialdehyde (MDA) content was measured by using lipid peroxidation MDA assay kit (Beyotime, S0131). OS cells were plated at a density of 1 × 10^5^ cells/mL in 6-well cell culture plates and allowed to attach. The following day, the cells were treated with PEITC for 24 h. After treatment, the cells were lysed by RIPA lysis buffer (Beyotime, P0013B) and centrifuged. The test solution was added to the supernatant. MDA level was determined by a Synergy HT multimode microplate reader (BioTek, Winooski, Vermont, USA) at 535 nm and normalized to protein concentration.

### 2.12. Measurement of GSH

The levels of total glutathione and oxidized glutathione were measured by using the GSH/GSSG assay kit (Beyotime, S0053). In brief, OS cells were seeded at a density of 1 × 10^5^ cells/mL in 6-well cell culture plates and allowed to attach. The following day, the cells were treated with PEITC for 24 h. The cells were then washed with cold PBS and lysed by two cycles of freezing and thawing. The samples were then centrifuged at 10,000 × g for 10 min, and the supernatant was collected for determination of total glutathione and oxidized glutathione (GSSG). The intracellular GSH was measured by a Synergy HT multimode microplate reader (BioTek, Winooski, Vermont, USA) at 412 nm. The level of reduced GSH was obtained by subtracting GSSG from the total glutathione and normalized to protein concentration.

### 2.13. Morphological Observation of Mitochondria and Nuclei

The mitochondria and nuclei were labeled by MitoTracker Green (Thermo, M7514) and Hoechst 33342 (Beyotime, C1022), respectively. In brief, OS cells were seeded at a density of 7.5 × 10^4^ cells/mL in 24-well cell culture plates and allowed to attach. The following day, the cells were treated with PEITC for 24 h. After PEITC treatment, the cells were washed with PBS and stained with 20 nM MitoTracker Green and Hoechst 33342 solution for 30 min at 37°C protected from light, respectively. The cells were washed with PBS again and imaged by an MD IL HC inverted fluorescence microscope (Leica, Wetzlar, Germany).

### 2.14. Measurement of Mitochondrial Transmembrane Potential

The mitochondrial transmembrane potential was measured with JC-1 fluorescent probe (Beyotime, C2006). In brief, OS cells were seeded at a density of 1 × 10^5^ cells/mL in 6-well cell culture plates and allowed to attach. The following day, the cells were treated with PEITC for 24 h. After PEITC treatment, the cells were collected, washed with PBS, and incubated with JC-1 solution for 20 min at 37°C. The stained cells were washed with PBS and analyzed by a FACSCalibur flow cytometer (BD Biosciences, San Jose, CA, USA).

### 2.15. Detection of Acidic Vesicular Organelles Formation

The formation of intracellular acidic vesicular organelles was observed by using acridine orange (AO, Thermo, A3568). In brief, OS cells were seeded at a density of 7.5 × 10^4^ cells/mL in 24-well cell culture plates and allowed to attach. The following day, the cells were treated with PEITC for 24 h. Then, the cells were washed with PBS and stained with 10 *μ*M AO solution for 15 min at 37°C protected from light. After discarding the AO solution, the cells were washed again with PBS and immediately observed with an MD IL HC inverted fluorescence microscope (Leica, Wetzlar, Germany).

### 2.16. Detection of Lysosomes

Lysosomes were observed by using LysoTracker Red (Beyotime, C1046). In brief, OS cells were seeded at a density of 7.5 × 10^4^ cells/mL in 24-well cell culture plates and allowed to attach. The following day, the cells were treated with PEITC for 24 h. Then, the cells were washed with PBS and incubated with 500 nM lysosome staining solution for 3 h at 37°C protected from light. After washing with by PBS, the cells were observed with an MD IL HC inverted fluorescence microscope (Leica, Wetzlar, Germany).

### 2.17. Western Blotting Analysis

OS cells were seeded at a density of 1 × 10^5^ cells/mL in 6-well cell culture plates and allowed to attach. The following day, the cells were treated with PEITC at concentrations of 1 *μ*M, 15 *μ*M, and 30 *μ*M for 20 h or 30 *μ*M PEITC for 4 h, 12 h, 24 h, and 48 h. Then cultured cells were washed with cold PBS and lysed with RIPA lysis buffer containing 1% protease/phosphatase inhibitors (Cwbiotech, Beijing, China) for 20 min on ice. Cell lysates were centrifuged at 12,000 × g for 10 min, and the supernatant was collected. Protein concentrations were quantified using a BCA protein assay kit (Beyotime, P0012S) according to the manufacture's instruction. Equal amounts of protein samples were separated by SDS-PAGE and transferred onto PVDF membrane. The membranes were blocked with 5% skimmed milk in TBST at room temperature for 3 h and then incubated with primary antibodies at 4°C for overnight. Antibodies against *β*-actin (4967), Beclin1 (3495), p62 (5114), ERK (4348S), p-ERK (8544S), JNK (9252S), p-JNK (9251S), p38 (11451S), p-p38 (4092S), and IRP2 (37135) were purchased from Cell Signaling Technology (Danvers, MA, USA). Antibodies against Caspase3 (AC030), C-caspase3 (AC033), and C-PARP (AF1567) were purchased from Beyotime Biotechnology (Shanghai, China). Antibodies against Bcl2 (18858), Bax (182733), LC3B (48394), Cdc2 (28439), FTH1 (75972), NCOA4 (86707), and GPx4 (185689) were purchased from Abcam (Cambridge, MA, USA). Antibodies against TfR1 (13-6800), FPN (PA5-22993), and DMT1 (PA5-35136) were purchased from Thermo Fisher Scientific (Waltham, MA, USA). After washed with TBST, the membranes were incubated with an HRP-conjugated secondary antibody (Beyotime, A0216 and A0208) at room temperature for 2 h. Each band was visualized by BeyoECL Moon (Beyotime, P0018FM) on a T5200 multi automatic fluorescence/chemiluminescence imaging system (Tanon).

### 2.18. RNA Isolation and qRT-PCR Analysis

Total RNA from the PEITC treatment groups was extracted by Trizol (Invitrogen, Carlsbad, CA, USA). mRNA was reverse-transcribed into cDNA using HiScript II Q RT SuperMix (Vazyme Biotech Co., Ltd, Nanjing, China, R222-01). The relative mRNA expression levels of *TFRC*, *SLC11A2*, *FTH1*, *SLC40A1*, and *IRP2* were determined using q-PCR with the cDNA template and ChamQ SYBR qPCR Master Mix (Vazyme Biotech, Q311-02) in a CFX96 Touch qPCR System (BioRad, Hercules, CA, USA). The sequences of forward and reverse primers of these genes are as follows: *GADPH*, forward: 5′ TGC ACC ACC AAC TGC TTA G 3′, reverse: 5′ GGA TGC AGG GAT GAT GTT C 3′; *TFRC*, forward: 5′ ACC ATT GTC ATA TAC CCG GTT CA 3′, reverse: 5′ CAA TAG CCC AAG TAG CCA ATC AT 3′; *SLC11A2*, forward: 5′ GTG GTC AGC GTG GCT TAT CT 3′, reverse: 5′ AGC TGT CAA TCC CAG ATG GC 3′; *FTH1*, forward: 5′ CTG CAC AAA CTG GCC ACT GA 3′, reverse: 5′ TCA CGT GGT CAC CCA ATT CTT 3′; *SLC40A1*, forward: 5′ CTA CTT GGG GAG ATC GGA TGT 3′, reverse: 5′ CTG GGC CAC TTT AAG TCT AGC 3′; and *IRP2*, forward: 5′ GAC GCC CCA AAA GCA GGA TA 3′, reverse: 5′ TCG TAC AGC AGC TTC CAA CA 3′. The primers of these genes are synthesized by Sangon Biotech Co., Ltd. (Shanghai, China). The data were calibrated to *GAPDH* and analyzed via the 2^−ΔΔCt^ method.

### 2.19. OS Xenograft Model

All animal experimental procedures conducted in accordance with protocols were approved by the Northwestern Polytechnical University Animal Care and Use Committee (No. 201900017). All efforts were made to reduce animal suffering. Four-week-old male BALB/c nude mice (16 g–18 g) were purchased from Beijing Vital River Laboratory Animal Technology Co., Ltd. (Beijing, China). They were maintained under specific pathogen-free regulated environment with a 12 h light/dark cycle and supplied with food and water *ad libitum*. Five mice shared one cage. After one week's adaptation, MNNG/HOS human OS cells were harvested, and a total of 1 × 10^7^ MNNG/HOS cells in 100 *μ*L of mixture of PBS and Matrigel (1 : 1) (BD, 356234) were injected subcutaneously into the right flank of the mouse. When the mice developed the tumor volume about 100 mm^3^, they were randomized into two groups of 8–10 mice per group and treated as follows: 10% sesame-seed oil with saline in control group and 30 mg/kg PEITC (PEITC in 10% sesame-seed oil with saline) in treated group. Mice were intragastrically administrated at afternoon daily for 24 days. The tumor size and body weight were measured every 3 days. The tumor volume was calculated in accordance with the formula: length × width^2^/2. After the final treatment of the experiment, all the mice were euthanized with isoflurane and then sacrificed by cervical dislocation. Tumor tissues were harvested, weighted, then snap-frozen in liquid nitrogen, and stored at -80°C. The harvested samples were used for Western blotting and histopathological analysis.

### 2.20. Histopathology

Formalin-fixed tissue samples were embedded in paraffin, and 4 *μ*m sections were cut. Primary tumor sections were stained with H&E for routine histological examinations and morphometric analysis. The sections were visualized by using an Eclipse 80i fluorescence microscope (Nikon, Tokyo, Japan).

### 2.21. Statistical Analysis

Statistical analysis was performed by using GraphPad Prism software, and the data were displayed as mean ± SD. One-way analyses of variance or Student's *t*-test was used for statistical analysis. Significance was defined at ^∗^*P* < 0.05, ^∗∗^*P* < 0.01, and ^∗∗∗^*P* < 0.001 versus control group.

## 3. Results

### 3.1. PEITC Reduced Viability and Induced Cell Death in Human OS Cells

**Figure 1:**
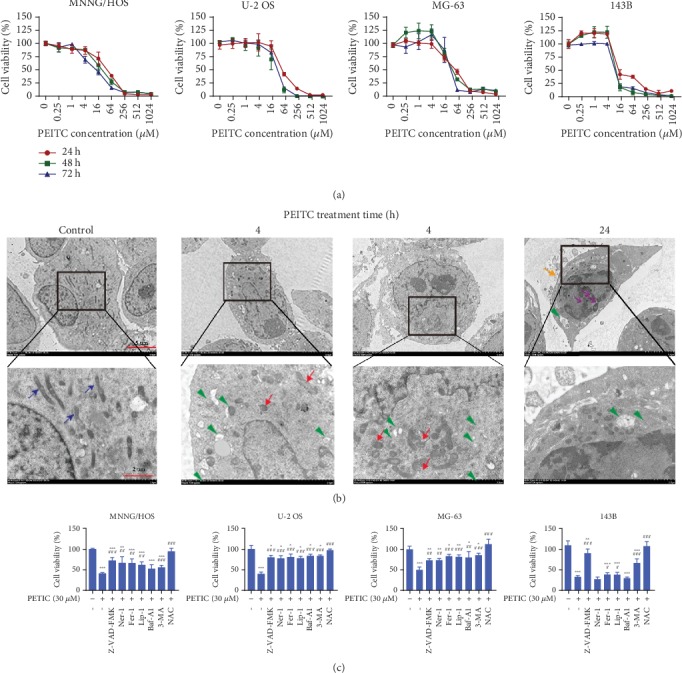
PEITC reduced viability and induced cell death in human OS cells. (a) Viability of MNNG/HOS, U-2 OS, MG-63, and 143B cells treated with series concentrations of PEITC for 24 h, 48 h, and 72 h. The data were presented as mean ± SD (*n* = 4). (b) The subcellular structural changes in MNNG/HOS OS cells treated with 30 *μ*M PEITC for 4 h, 12 h, and 24 h analyzed by TEM. Blue arrows indicated intact mitochondria. Green triangles indicate autophagic vacuoles containing intact and degraded cellular debris. Red arrows indicate round and shrunken mitochondria. Yellow arrows indicate a blebbed membrane. Purple arrows indicate nuclear fragmentation. (c) Viability of MNNG/HOS, U-2 OS, MG-63, and 143B cells treated with PEITC in the presence of 50 *μ*M z-VAD-FMK, 50 *μ*M Ner-1, 10 *μ*M Fer-1, 100 nM Lip-1, 1 *μ*M Baf-A1, 10 mM 3-MA, or 1 mM NAC for 24 h. The data were presented as mean ± SD (*n* = 4). ^∗^*P* < 0.05, ^∗∗^*P* < 0.01, and ^∗∗∗^*P* < 0.001 versus control group. ^#^*P* < 0.05, ^##^*P* < 0.01, and ^###^*P* < 0.001 versus PEITC treatment group.

CCK-8 assay was used to assess the viability of human OS cells after treatment with serial concentrations of PEITC for different time periods. The results indicated that PEITC significantly inhibited the viability of human OS cells in concentration- and time-dependent manners (Figure 1(a)). After PEITC treatment, MNNG/HOS OS cells exhibited obvious subcellular structure changes in mitochondria, nuclei, and autophagic vacuoles by transmission electron microscopy (TEM) imaging (Figure 1(b)). The mitochondria of MNNG/HOS OS cells became into round, shrunk, and dilated shape with reduced/disappeared cristae at 4 h of PEITC treatment as compared with the elongated ones in the control group. There were double-membrane vacuoles with undigested contents and single-membrane vacuoles with degradation of contents in PEITC-treated MNNG/HOS OS cells. Chromatin condensation, nuclear fragmentation, and blebbed membrane appeared when PEITC treatment lasted for 24 h. MNNG/HOS OS cells displayed obvious subcellular structural characteristics in mitochondria, autophagic structures, and nuclei, indicating the possible onset of ferroptosis, autophagy, and apoptosis by PEITC treatment.

To confirm the cytotoxic effects of PEITC on human OS cells, we investigated whether the reduced viability was due to the possibility of triggering cell death. The viability of human OS cells treated with PEITC in the presence of caspase inhibitor z-VAD-FMK, RIP1 kinase inhibitor Ner-1, lipid ROS scavenger Fer-1 and Lip-1, H^+^-ATPase inhibitor Baf-A1, phosphoinositide 3-kinase (PI3K) inhibitor 3-MA, or an antioxidant NAC were examined. The results indicated that apoptosis inhibitor, necroptosis inhibitor, autophagy inhibitors, and ferroptosis inhibitors partially rescued cell death induced by PEITC, whereas antioxidant NAC totally rescued the reduced viability induced by PEITC in OS cells (Figure 1(c)). Cell death inhibitors partly protected the cells against cell death, which highlighted that apoptosis, necropoptosis, autophagy, and ferroptosis were triggered in human OS cells by PEITC treatment.

### 3.2. PEITC Inhibited Proliferation of Human OS Cells

**Figure 2:**
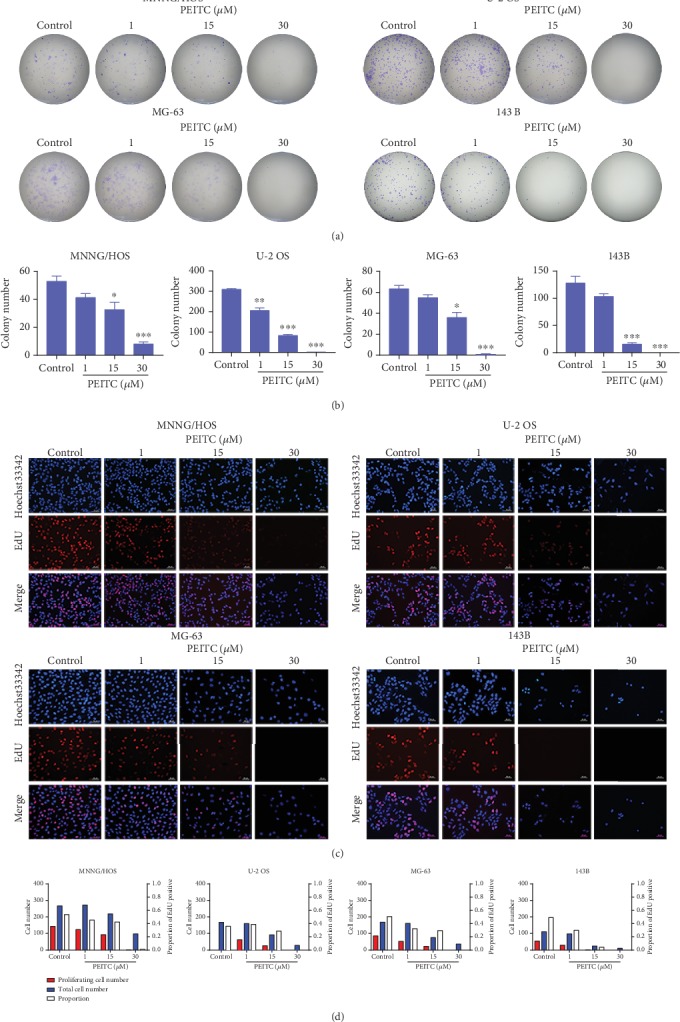
PEITC inhibited cell proliferation of human OS cells. (a) Colony formation assay of MNNG/HOS, U-2 OS, MG-63, and 143B cells treated with PEITC. (b) Quantitative analysis of the colonies in (a). The data were presented as mean ± SD (*n* = 3). ^∗^*P* < 0.05, ^∗∗^*P* < 0.01, and ^∗∗∗^*P* < 0.001 versus control group. (c) EdU staining assay of MNNG/HOS, U-2 OS, MG-63, and 143B cells treated with PEITC. (d) Quantitative analysis of EdU staining in (c).

To further verify the inhibitory effect of PEITC on the proliferative potential of human OS cells, both colony formation and EdU assays were conducted. The results demonstrated that PEITC exhibited concentration-dependent inhibitory effects on the proliferation of four human OS cell lines, and higher concentration of PEITC significantly reduced the colony formation capacity of human OS cells as compared with the control group (Figures 2(a) and 2(b)). As shown in Figures 2(c) and 2(d), after PEITC treatment, there displayed less Hoechst 33342 staining cells, less EdU-positive cells, and the proportion of EdU-positive cells were decreased. These results indicated that PEITC inhibited the proliferation of human OS cells.

### 3.3. PEITC Induced G_2_/M Cell Cycle Arrest in Human OS Cells

**Figure 3:**
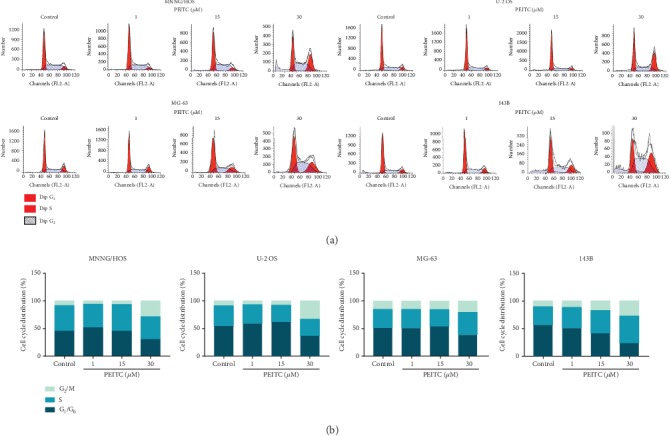
PEITC induced G_2_/M cell cycle arrest in human OS cells. (a) The cell cycle distribution of MNNG/HOS, U-2 OS, MG-63, and 143B cells after the indicated concentrations of PEITC treatment for 24 h by PI staining. (b) Quantitative analysis of cell cycle distribution in (a).

We speculated whether the inhibitory effects of PEITC on cell proliferation of human OS cells related to its effect on cell cycle progression. Results of cell cycle distribution showed that exposure of human OS cells to PEITC for 24 h led to increased proportion of cells in G_2_/M phase and a corresponding decrease in G_0_/G_1_ phase (Figures 3(a) and 3(b)).

### 3.4. PEITC Altered Iron Metabolism in Human OS Cells

**Figure 4:**
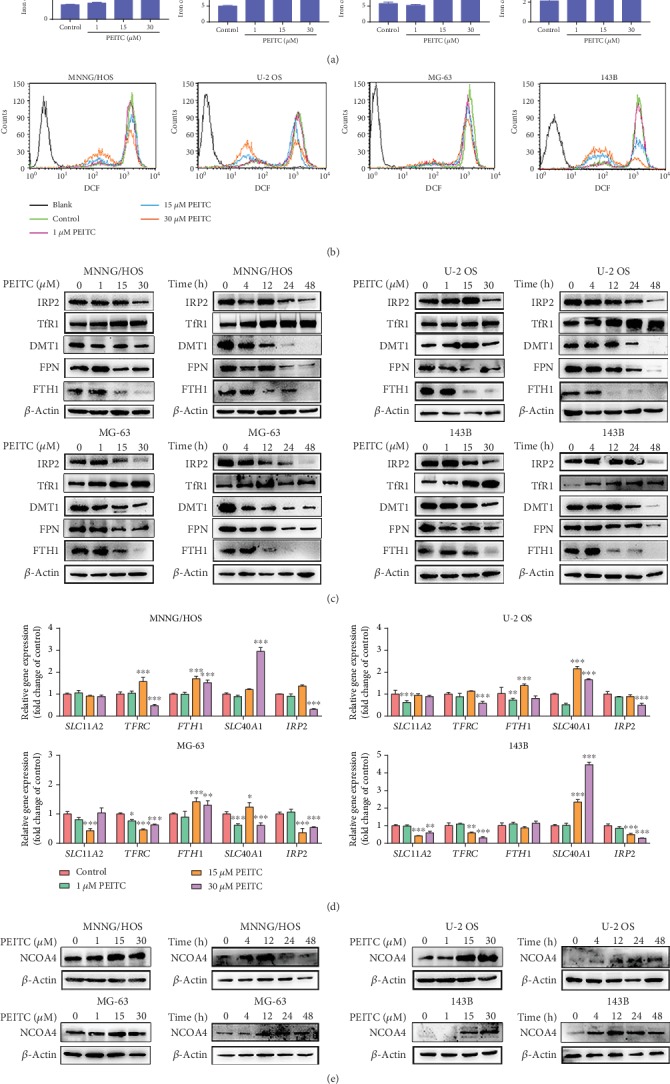
PEITC altered iron metabolism in human OS cells. (a) Total iron level in MNNG/HOS, U-2 OS, MG-63, and 143B cells after the indicated concentrations of PEITC treatment for 24 h by AAS. (b) The level of labile iron in MNNG/HOS, U-2 OS, MG-63, and 143B cells after the indicated concentrations of PEITC treatment for 24 h with Calcein-AM staining by flow cytometry analysis. (c) Protein expression levels of TfR1, DMT1, FTH1, FPN, and IRP2 in MNNG/HOS, U-2 OS, MG-63, and 143B cells treated with the indicated concentrations of PEITC for 20 h or 30 *μ*M PEITC for 4 h, 12 h, 24 h, and 48 h. (d) mRNA levels of *TFRC*, *SLC11A2*, *FTH1*, *SLC40A1*, and *IRP2* in MNNG/HOS, U-2 OS, MG-63, and 143B cells after PEITC treatment for 24 h. (e) Protein expression levels of NCOA4 in MNNG/HOS, U-2 OS, MG-63, and 143B cells treated with the indicated concentrations of PEITC for 20 h or 30 *μ*M PEITC for 4 h, 12 h, 24 h, and 48 h. All data were presented as the means ± SD (*n* = 3). ^∗^*P* < 0.05, ^∗∗^*P* < 0.01, and ^∗∗∗^*P* < 0.001 versus control group.

Iron is an important element for life that participates in many cellular processions. Alterations in iron metabolism in tumors lead to tumor growth or death. We tested whether iron metabolism was altered in PEITC-treated human OS cells. First, we measured total iron content by AAS. The results indicated that PEITC significantly increased total iron level in four human OS cell lines (Figure 4(a)). Then, we analyzed the level of intracellular labile iron using Calcein-AM sensor. It can be seen from Figure 4(b), the number of cells with darken green fluorescence was increased accompanied with decreased number of cells with light green fluorescence as PEITC concentration increased in MNNG/HOS, U-2 OS, and 143B cells. MG-63 cells exhibited less evident changes in number of cells with darken green fluorescence. It concluded that after PEITC treatment, there was a shift in the cell number from cells with lower content labile iron toward cells with higher content of labile iron in MNNG/HOS, U-2 OS, and 143B cells.

Iron is tightly regulated by controlling iron uptake, storage, utilization, and efflux. We suspected whether alterations in iron metabolism regulators resulted in both elevated total iron level and the shifts in labile iron in human OS cells. The Western blotting showed that the expression of TfR1 was upregulated after PEITC treatment, and FPN, FTH1, and DMT1 were all downregulated (Figure 4(c)). The protein expression level of IRP2 was decreased in PEITC-treated human OS cells. IRP2 is an iron-dependent protein, which senses cellular labile iron by regulating the processes of iron import and export. The decreased expression levels of IRP2 indicated the increased level of labile iron.

To further explore the modulation mechanism of PEITC on iron metabolism in human OS cells, we detected mRNA expression levels of iron-regulatory genes. Interestingly, we found that the mRNA transcript levels of *FTH1* and *SLC40A1*both increased while the transcript levels of *IRP2*, *TFRC*, and *SLC11A2* all decreased in human OS cells after PEITC treatment (Figure 4(d)). Increase of labile iron led to increased translation of mRNAs of *FTH1* and *SLC40A1* and degradation of *TFRC* mRNA.

The increase of labile iron can be from TfR1-mediated iron import and/or ferritin degradation. Nuclear receptor coactivator 4 (NCOA4) is a cargo receptor which mediates ferritin degradation in the autophagosome in the ferritinophagy process. In Figure 4(e), NCOA4 level was increased and then decreased in human OS cells. FTH1 levels were lower in PEITC-treated group because of degradation and iron liberating from them. Therefore, these results clarified that the higher level of iron in PEITC-treated human OS cells was owing to high expression of TfR1, down expressions of FTH1 and FPN, which together leading to uptake of iron, degradation of iron storage proteins, and decrease in iron efflux.

### 3.5. PEITC Induced Oxidative Stress in Human OS Cells

**Figure 5:**
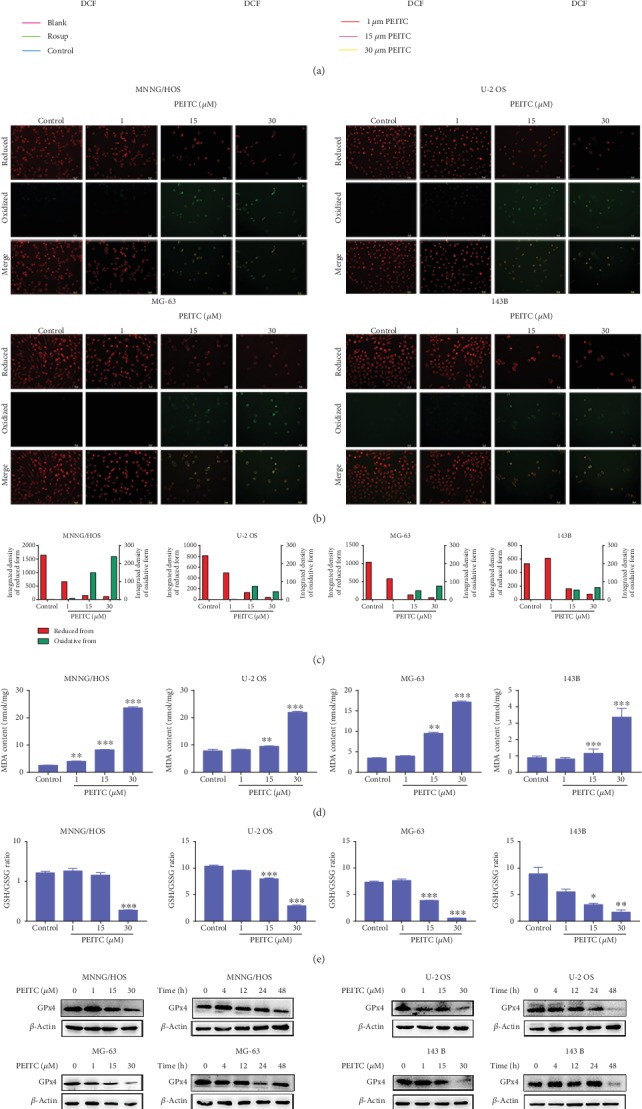
PEITC induced oxidative stress in human OS cells. (a) ROS level in MNNG/HOS, U-2 OS, MG-63, and 143B cells treated with the indicated concentrations of PEITC for 24 h by using a DCFH-DA sensor. (b) Lipid peroxidation level in MNNG/HOS, U-2 OS, MG-63, and 143B cells treated with the indicated concentrations of PEITC for 24 h by using a BODIPY 581/591 C11 sensor. (c) Quantitative analysis of lipid peroxidation in (b). (d) MDA level in MNNG/HOS, U-2 OS, MG-63, and 143B cells treated with the indicated concentrations of PEITC for 24 h. (e) GSH/GSSG ratio in MNNG/HOS, U-2 OS, MG-63, and 143B cells treated with the indicated concentrations of PEITC for 24 h. (f) GPx4 expression in MNNG/HOS, U-2 OS, MG-63, and 143B cells treated with the indicated concentrations of PEITC for 20 h or 30 *μ*M PEITC for 4 h, 12 h, 24 h, and 48 h. All data were presented as the mean ± SD (*n* = 3). ^∗^*P* < 0.05, ^∗∗^*P* < 0.01, and ^∗∗∗^*P* < 0.001 versus control group.

To validate whether PEITC caused oxidative stress in human OS cells, we tested the alterations of cytosolic ROS levels in OS cells treated by PEITC for 24 h with DCFH-DA sensor. After PEITC treatment, there appeared significant cellular ROS generation in MNNG/HOS, MG-63, and 143B cells (Figure 5(a)). Then, we measured lipid ROS by using BODIPY 581/591 C11. In Figures 5(b) and 5(c), PEITC treatment for 24 h increased the level of oxidized lipid ROS in human OS cells. In order to confirm whether ROS induced by PEITC exerted oxidative stress in human OS cells, we measured the level of MDA, which is a product generated from lipid peroxidation. Likewise, there was significant elevation in MDA corresponding to lipid peroxidation by PEITC treatment in human OS cells (Figure 5(d)).

Maintenance of cellular redox balance is essential for cell fate. GSH is the major cellular redox buffer, and GSH/GSSG ratio is an important indicator for the redox status in cells. We speculated that the excess ROS might be a result of the defect in the antioxidant system, which lacks the capability of ROS removal. Therefore, the effect of PEITC on the GSH antioxidant system was detected in human OS cells. The results demonstrated that PEITC significantly decreased GSH/GSSG ratio (Figure 5(e)). The expression level of GPx4 in human OS cells was decreased after PEITC treatment for at least 24 h (Figure 5(f)). In conclusion, we speculated that PEITC impaired cellular GSH antioxidant defense accompanied with reducing GSH/GSSG ratio and lowering GPx4 expression, which further led to ROS accumulation, lipid peroxidation, and oxidative stress in human OS cells.

### 3.6. PEITC Induced Mitochondria-Mediated Apoptosis in Human OS Cells

**Figure 6:**
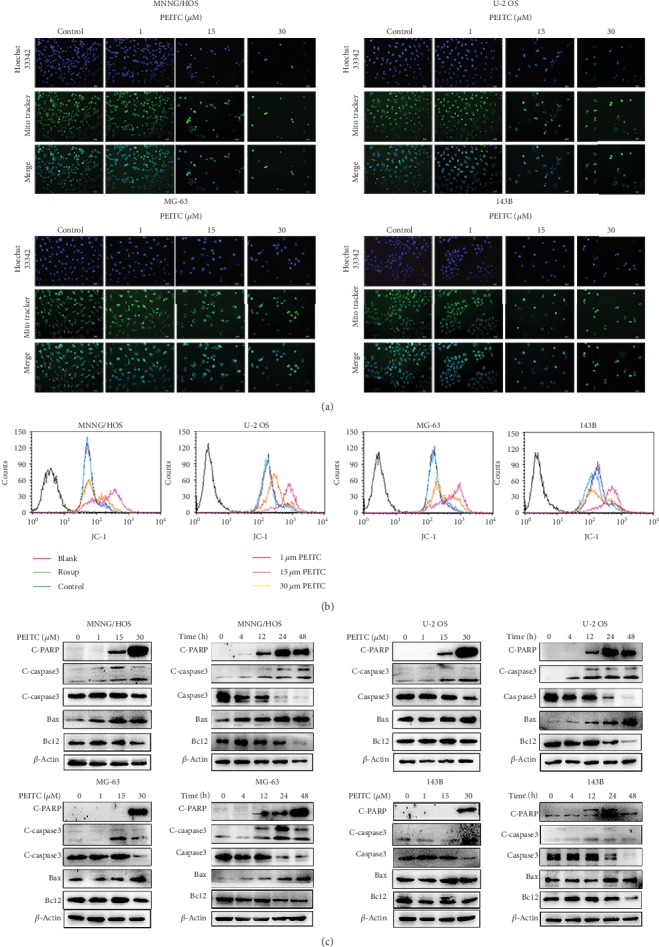
PEITC induced mitochondria-mediated apoptosis in human OS cells. (a) Mitochondrial and nuclear morphological changes in MNNG/HOS, U-2 OS, MG-63, and 143B cells treated with the indicated concentrations of PEITC for 24 h by MitoTracker Green/Hoechst 33342 staining. (b) Mitochondrial transmembrane potential in MNNG/HOS, U-2 OS, MG-63, and 143B cells treated with the indicated concentrations of PEITC for 24 h by using JC-1. (c) Protein expression levels of Caspase3, C-caspase3, Bcl2, Bax, and C-PARP in MNNG/HOS, U-2 OS, MG-63, and 143B cells treated with the indicated concentrations of PEITC for 20 h or 30 *μ*M PEITC for 4 h, 12 h, 24 h, and 48 h.

In order to, respectively, confirm the multiple cell death modalities induced by PEITC in human OS cells, we firstly detected the key events of apoptosis. MitoTracker Green and Hoechst 33342 labeling assays showed that there were more and more fragmented mitochondria accumulating around nuclei with intensified green fluorescence in four human OS cell lines after PEITC treatment as compared with those uniformly distributed in the control group (Figure 6(a)). To investigate whether alterations in mitochondrial membrane potential were involved in PEITC-induced cell death in human OS cells, JC-1 fluorescent probe was used. As shown in Figure 6(b), the higher concentration of PEITC, the more obviously decreased level of mitochondrial membrane potential with intensified green fluorescence, which indicating the increased proportion of human OS cells with depolarized mitochondria.

The decline in mitochondria membrane potential is a hallmark of early apoptosis and an indicative of mitochondrial outer membrane permeabilization which is crucial for the release of apoptosis factors. Further down, we performed Western blotting to explore the molecular mechanism of PEITC-induced apoptosis. As shown in Figure 6(c), higher concentration of PEITC increased the level of pro-apoptotic protein Bax and lowered the level of antiapoptotic protein Bcl2 in human OS cells. Caspases are the executors of intrinsic apoptosis pathway. The expression of C-caspase3 was upregulated. The substrate of activated caspase, C-PARP, was measured, and the results indicated that there was increased expression of C-PARP in four human OS cell lines. The overall results revealed that PEITC induced caspase cascade apoptosis through mitochondria-mediated pathway in human OS cells.

### 3.7. PEITC Induced Autophagy in Human OS Cells

**Figure 7:**
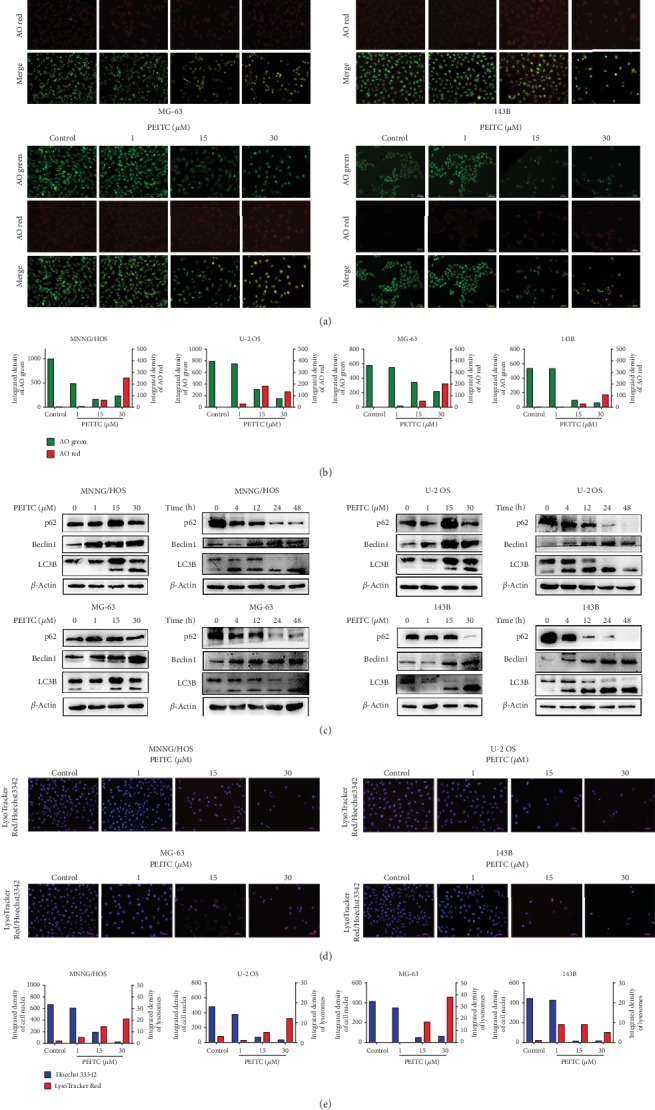
PEITC induced autophagy in human OS cells. (a) Acidic vacuole organelles in MNNG/HOS, U-2 OS, MG-63, and 143B cells treated with the indicated concentrations of PEITC for 24 h by AO staining. (b) Quantitative analysis of AO red fluorescence and AO green fluorescence in (a). (c) Protein expression levels of Beclin1, LC3B, and p62 in MNNG/HOS, U-2 OS, MG-63, and 143B cells treated with the indicated concentrations of PEITC for 20 h or 30 *μ*M PEITC for 4 h, 12 h, 24 h, and 48 h. (d) Lysosomes in MNNG/HOS, U-2 OS, MG-63, and 143B cells treated with the indicated concentrations of PEITC for 24 h by using a LysoTracker Red sensor. (e) Quantitative analysis of lysosomes in (d).

Since lysosomal inhibitor Baf-A1 and autophagy inhibitor 3-MA partially protected human OS cells from PEITC-induced cell death, we further investigated whether autophagic cell death was also triggered. The formation of acidic vesicular organelles is one of the characteristics of autophagy. In Figures 7(a) and 7(b), PEITC treatment caused significantly large proportion of cells forming acidic vesicular organelles which emitted red fluorescence. It further indicated that PEITC may cause efficient autophagy process in human OS cells. Meanwhile, we examined the typical markers that represent autophagic process. Beclin1 initiates autophagic process by binding to PI3K. It was clear that treatment with PEITC enhanced Beclin1 expression in human OS cells (Figure 7(c)). We also observed significant conversion of LC3I to LC3II in human OS cells as PEITC concentration and treatment time increased. In MNNG/HOS, U-2 OS, and 143B cells, the conversion of LC3I to LC3II mainly occurred as early as 4 h of PEITC treatment and lasted to 12 h, indicating the enhanced accumulation of autophagosomes in this period of time. After 24 h, there was only evident accumulation of LC3II in these three OS cell lines. As for MG-63, the conversion also happened at 4 h, but there was still the conversion of LC3I to LC3II in the presence of PEITC for 24 h. The higher level of LC3II reflected the accumulation of autophagosomes. In addition to LC3II accumulation, p62 protein was also detected. p62 binds to LC3 and then is degraded in autolysosomes. The change in p62 level indicates the autophagic flux. It suggested that p62 significantly decreased along with time in human OS cells in response to PEITC treatment. Last, we detected lysosomes by staining human OS cells with LysoTracker Red. As seen in Figures 7(d) and 7(e), the ratio of LysoTracker Red-positive cells was higher in the PEITC-treated group, especially in MG-63 cells. On all accounts, PEITC treatment induced autophagy in human OS cells.

### 3.8. PEITC Activated the MAPK Signaling Pathway in Human OS Cells

**Figure 8:**
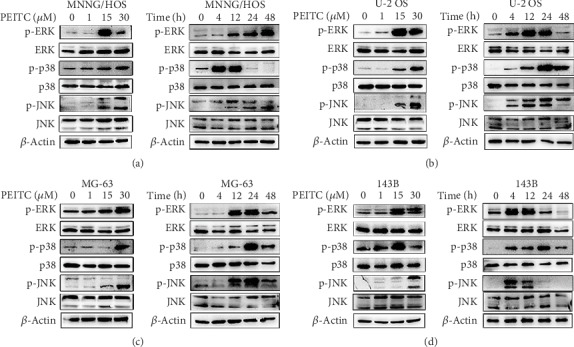
PEITC activated the MAPK signaling pathway in human OS cells. (a–d) Phosphorylation levels of ERK, p38, and JNK in MNNG/HOS, U-2 OS, MG-63, and 143B cells treated with the indicated concentrations of PEITC for 20 h or 30 *μ*M PEITC for 4 h, 12 h, 24 h, and 48 h.

It has been reported that MAPK signaling pathway plays a role in the pharmacological effects of some ITCs; thus, it would be interesting to detect the effects of PEITC on it in human OS cells. As shown in Figures 8(a)–8(d), PEITC significantly activated ERK, p38, and JNK in human OS cells after PEITC treatment. The extents of MAPKs being activated were different among four human OS cell lines. The phosphorylation of ERK appeared as early as 4 h after PEITC treatment; the effect extended to 24 h and gradually weakened at 48 h in U-2 OS and 143B cells. With respect to MNNG/HOS and MG-63 cells, the phosphorylation of ERK was detected at 12 h, substantially reduced in MG-63 cells and up to the higher level in MNNG/HOS cells at 48 h treatment, respectively. The activation of p38 was stronger at 4 h after PEITC treatment, sustained to 12 h, and then the activation effects lost in MNNG/HOS cells. In both U-2 OS and 143B cells, PEITC treatment led to p-p38 occurring at 4 h; the phosphorylated effect gradually increased to a much stronger level at 24 h and thereafter significantly decreased. To MG-63 cells, p-p38 occurred at 12 h, maximized at 24 h, and gradually decreased later. JNK was activated as early as 4 h after PEITC treatment in U-2 OS and 143B cells while at 12 h in MNNG/HOS and MG-63 cells. Phosphorylation of JNK was still underway at 48 h in MNNG/HOS, MG-63, and U-2OS cells except for that in 143B cells, which was already over at 24 h. These above results indicated that PEITC activated MAPK signaling pathway in four human OS cell lines with different responses.

### 3.9. ROS Acted as an Upstream Signal in PEITC-Induced Cell Death in Human OS Cells

**Figure 9:**
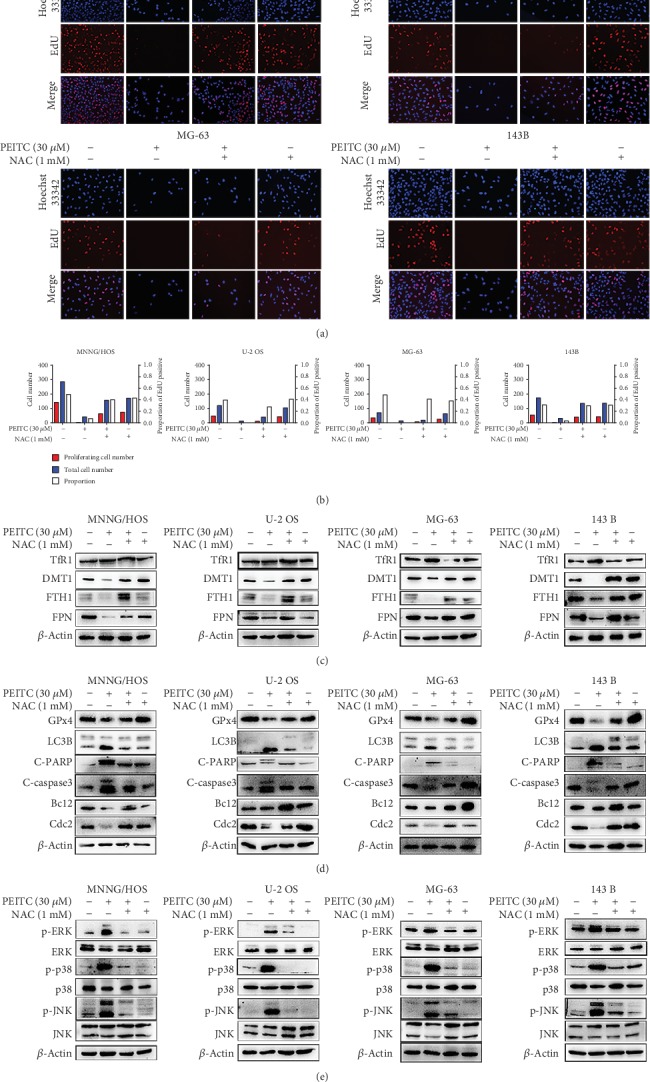
PEITC induced cell death via ROS generation in human OS cells. (a) EdU staining assay of MNNG/HOS, U-2 OS, MG-63, and 143B cells treated with 30 *μ*M PEITC in the presence or absence of NAC for 24 h. (b) Quantitative analysis of EdU staining in (a). (c) Protein expression levels of TfR1, DMT1, FTH1, and FPN in MNNG/HOS, U-2 OS, MG-63, and 143B cells treated with 30 *μ*M PEITC in the presence or absence of NAC for 24 h. (d) Protein expression levels of GPx4, LC3B, C-PARP, C-caspase3, Bcl2, and Cdc2 in MNNG/HOS, U-2 OS, MG-63, and 143B cells treated with 30 *μ*M PEITC in the presence or absence of NAC 24 h. (e) Phosphorylation levels of ERK, p38, and JNK in MNNG/HOS, U-2 OS, MG-63, and 143B cells treated with 30 *μ*M PEITC in the presence or absence of NAC for 24 h.

To test whether ROS acted as an upstream signal in PEITC-induced cell death in human OS cells, we performed serials of assays by cotreatment with NAC. As seen in Figures 9(a) and 9(b), PEITC-induced weak proliferation potential was significantly inhibited when co-treated with NAC in four human OS cell lines. The decreased expressions of FPN, DMT1, and FTH1 after PEITC treatment were upregulated when co-treated with NAC in human OS cells. PEITC-induced increased expression of TfR1 was reduced when NAC was co-used with PEITC (Figure 9(c)). To investigate whether PEITC-induced ROS generation exhibited role in affecting cell growth and inducing cell death in human OS cells, we detected the typical markers for cell cycle progression, ferroptosis, apoptosis, and autophagy. In Figure 9(d), NAC altered the PEITC-induced drop in the expressions of Cdc2, Bcl2, and GPx4 and increase in LC3B, C-caspase, and C-PARP. Enlightened by the PEITC-activated MAPK signaling pathway, we also checked whether the activation of MAPKs was a result of ROS generation. From Figure 9(e), we found that PEITC treatment actually caused ROS-dependent MAPK signaling pathway activation because NAC inhibited phosphorylations of ERK, p38, and JNK, which were activated by PEITC in human OS cells. Collectively, these results implied that PEITC-induced ROS generation played a critical role in inducing inhibition on cell proliferation, triggering cell death, and activating MAPKs signaling pathway in human OS cells.

### 3.10. PEITC Exerted an Antitumor Effect in Xenograft OS Mice

**Figure 10:**
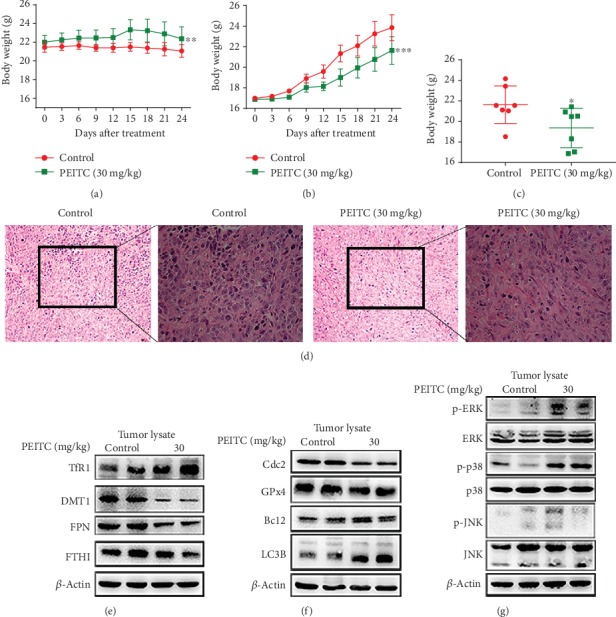
PEITC suppressed OS growth *in vivo*. MNNG/HOS cells were injected subcutaneously into the right flank of the male BALB/c nude mice. One week after the OS xenograft mouse model was established, the mice were randomly divided into two groups and, respectively, administrated with 10% sesame seed oil and 30 mg/kg PEITC once daily for 24 consecutive days. (a) Body weight change of the two groups. (b) Volume change of OS tissues of the two groups. Data were calculated by the formula: volume = length × width^2^ × 0.5. (c) Weight of OS tissues from the two groups. (d) H&E staining analysis of tumor tissues (200× and 400×). (e) The protein expression levels of TfR1, DMT1, FTH1, and FPN in tumor tissues. (f) The protein expression levels of LC3B, C-caspase3, GPx4, and Cdc2 in tumor tissues. (g) Phosphorylation levels of ERK, p38, and JNK in tumor tissues. All data were presented as the means ± SD (*n* = 7). ^∗^*P* < 0.05, ^∗∗^*P* < 0.01, and ^∗∗∗^*P* < 0.001 versus control group.

The *in vivo* anti-OS effects were conducted by intragastrical administration of PEITC with a dose of 30 mg/kg for consecutive 24 days in nude mice transplanted with MNNG/HOS cells. The results showed that PEITC-administrated group exhibited significant increase in body weight compared with the control group (Figure 10(a)). Importantly, it was worth noting that PEITC treatment did not cause weight loss in mice with OS cells, which may indicate the lower toxicity of PEITC at a dose of 30 mg/kg. Next, we compared the tumor volume and tumor weight between the two groups. The results in Figures 10(b) and 10(c) revealed that PEITC delayed tumor growth with respect of both decreased tumor volume and lowered tumor weight. H&E staining assay suggested that PEITC treatment induced cell death in OS tissues (Figure 10(d)). In Figure 10(e), the tumor lysates in PEITC treatment group exhibited decreased FPN and DMT1 expressions, while TfR1 expression was upregulated as compared with control group. The protein expression levels of cell cycle progression related protein Cdc2, antioxidant enzyme GPx4, apoptotic related protein Bcl2 and Bax, and autophagic marker LC3B after PEITC administration were consistent with the previous results in *in vitro* (Figure 10(f)). As seen in Figure 10(g), significant activations of ERK and p38 occurred in PEITC-administrated group. Together, PEITC at a dose of 30 mg/kg exhibited lower system toxicity with significant tumor inhibitory effect in OS mice through activating cell death signaling pathways.

## 4. Discussion

**Figure 11:**
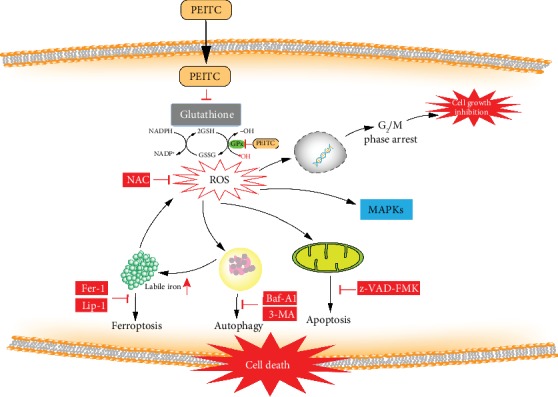
A schematic diagram of the effect of PEITC on human OS cells. PEITC induced G_2_/M cell cycle arrest, altered iron metabolism, activated the MAPK signaling pathway, and triggered ferroptosis, autophagy, and apoptosis in human OS cells through GSH depletion and ROS generation.

PEITC, one of the main active compounds in cruciferous vegetables, is the most promising antitumor ITCs both in preclinical models and clinical trials [18–20]. In the present study, we showed that PEITC reduced cell viability, inhibited proliferation, caused G_2_/M cell cycle arrest, altered iron metabolism, induced GSH depletion, generated ROS, activated MAPK signaling pathway, and triggered multiple cell death modalities, mainly ferroptosis, apoptosis, and autophagy, in human OS cells (Figure 11).

To date, most studies have reported that PEITC induces apoptosis in various cancers. PEITC induces G_2_/M cell cycle arrest and apoptosis in oral squamous cell carcinoma cells with various p53 statuses [21]. PEITC decreases antiapoptotic protein expressions and increased pro-apoptotic protein expressions in pancreatic cancer cells [22]. PEITC inhibits ovarian cancer cells by activating caspases and MAPK signaling pathway [23]. Moreover, PEITC combined with other agents also induces ferroptosis in pancreatic cancer [24]. The possible antitumor mechanisms of PEITC include GSH depletion, G_2_/M cell cycle arrest, mitochondria dysfunction, and MAPKs activation [25, 26].

In our study, blocking apoptosis with z-VAD-FMK did not totally prevent the cytotoxic effect of PEITC on four human OS cell lines. Similarly, inhibitor of necroptosis (Ner-1), inhibitors of ferroptosis (Fer-1 and Lip-1), and inhibitors of autophagy (Baf-A1 or 3-MA) all partially prevented the reduced viability after PEITC treatment. Therefore, we speculated that PEITC may induce multiple cell death modalities in human OS cells. The conclusion was further supported by serials of experimental evidence: (1) PEITC caused shrunk mitochondria, accumulated labile iron, induced GSH depletion, GPx4 inhibition, and lipid peroxidation. The main feature of ferroptosis is iron-dependent accumulation of lipid peroxidation that is morphologically, biochemically, and genetically different from apoptosis, autophagy, and necroptosis [27]. Therefore, it hinted that ferroptosis was initiated in PEITC-treated human OS cells. (2) PEITC caused condensed chromatin, depolarization of mitochondria membrane potential, modulation of apoptosis related proteins, activation of caspase, and cleavage of PARP. These results proved that PEITC triggered mitochondria-mediated apoptosis in human OS cells. (3) PEITC promoted the formation of acid vesicle organelles, enhanced LC3 conversion, increased Beclin1 expression, decreased p62 expression, and promoted autophagosome accumulation. The alterations suggested autophagy was also triggered in human OS cells. Together, above results supported that PEITC induced multiple cell death modalities, including ferroptosis, autophagy, and apoptosis, in human OS cells.

The cell death pathways are generally defined by biochemical and morphological alterations, whereas different cell death modalities display mixed characteristics and share some key regulatory proteins, suggesting mechanism underlying the regulation of different cell death pathway may be connected [28, 29]. Ferroptosis promotes the activation of the autophagic process for the degradation of ferritin and thus the accumulation of cellular labile iron [30]. NCOA4 acts as a cargo receptor that selectively targets ferritin to the autophagosome. Indeed, the induction of ferroptosis is dependent on the trigger of autophagy [31]. In our study, the decreased FTH1 expression accompanied with alteration on NCOA4 expression, which resulted in elevated labile iron liberating from ferritin. Meanwhile, autophagic marker LC3 conversion appeared in human OS cells as earlier as 4 h treatment of PEITC. Furthermore, evidence showed that ferroptosis is independent of apoptosis [32]. The initiation of ferroptosis proceeds from the release and activation of proapoptotic effectors [33]. Mitochondria became shrunken and damaged at the early stage of PEITC treatment while fragmentation and margination of chromatin appeared in the later time. It may indicate that ferroptosis is an early event, and apoptosis happened relatively later in PEITC-treated human OS cells.

ROS has dual functions in cellular physiology depending on their levels [34]. Cancer cells usually have higher ROS levels in order to sustain their malignant proliferation, whereas the higher endogenous ROS levels make them more susceptible to oxidative stress [35]. Alteration in the redox system and dysfunction in the antioxidant system might be useful in improving the drug resistance and the anticancer effects [36]. In previously published studies, PEITC treatment generates cellular ROS and subsequently induced cell death in many cancers [37, 38]. In our study, NAC totally reversed the cytotoxic effect of PEITC exerted on human OS cells. PEITC treatment increased cellular ROS and the levels of lipid peroxidation and its product MDA. Importantly, the antioxidant system was impaired as for PEITC treatment led to significant depletion of GSH and inhibition on GPx4 expression. The imbalance between the antioxidant system and ROS production made human OS cells more sensitive to oxidative stress. In addition, PEITC cotreated with NAC altered the effects of PEITC exerted on cell proliferation, cell cycle, iron metabolism, cell death pathway, and MAPKs. The disruption of intracellular redox homeostasis and irreversible peroxidation of lipids by PEITC treatment brought to the activation of cell death signals and inhibition on survival signals in human OS cells.

Iron is important for many cellular processes, and the abnormality in iron metabolism also causes cell death [39]. Iron metabolism is tightly controlled by a set of iron-dependent proteins and divided into the processes of iron intake, utilization, storage, and efflux. TfR1 and DMT1 mediate the intake of transferrin-bound iron and non-transferrin-bound iron; intracellular iron can be stored in ferritin for reducing the oxidative stress by excess labile iron; iron is exclusively exported by FPN [40]. In our study, we found that PEITC upregulated TfR1 and downregulated FTH1, FPN, and DMT1, leading to the elevations of both total iron and labile iron. In PEITC-induced high labile iron condition, the mRNAs of genes that lower the content of labile iron, such as *FTH1* and *SLC40A1*, were translationally activated. Meanwhile, the mRNAs of genes that facilitate iron uptake, such as *TFRC* and *SLC11A2*, were translationally degraded. There is a coordinated feedback control between the labile iron and iron metabolism genes, which is tightly regulated [41]. Labile iron can generate ROS through Fenton reaction or oxidation [42]. PEITC treatment increased labile iron through altering iron metabolism process and thus putting human OS cells in a situation that was more prone to oxidative stress. Except for the disruption on GSH antioxidant system, the effect on iron metabolism is also an important effector for PEITC-induced oxidative stress.

Previous studies indicate that oral gavage administration of PEITC (12 *μ*M PEITC/day, 5 days/week) suppresses the growth of pancreatic cancer cells in xenograft animal models, or diet consumption of PEITC (3 *μ*M PEITC/g/diet, 19 weeks) suppresses prostate cancer in a transgenic mouse model with no significant signs of organ toxicity [22, 43]. PEITC has been demonstrated having significant pharmacological effects in anticancer research *in vivo*. So far, there is no report concerning the effects of PEITC on osteosarcoma animal models. In our study, intragastrical administration of PEITC (30 mg/kg/d) exhibited lower system toxicity and still with significant tumor inhibitory effect in OS xenograft mice through altering iron metabolism, activating MAPKs, and triggering cell death signaling pathways. Moreover, PEITC is proved to exhibit 7.7% inhibitory effects on metabolic activation of lung carcinogen in cigarette smokers in clinical trials [13]. As for the efficacy in both preclinical and clinical trials, PEITC needs to be in-depth investigated for the potential clinical uses. Studies also show that a combination of PEITC with Adriamycin or Platinum can synergistically enhance the pharmacological effects of the first-line anticancer drugs and reverse the drug resistance [44, 45]. Therefore, it is of great implications to further explore the effects of PEITC when co-using with clinical anticancer drugs on osteosarcoma.

## 5. Conclusions

Altogether, ROS-inducing strategy can be considered as a promising therapeutic avenue in cancer treatment. PEITC triggered not one form of cell death modality but multiple cell death modalities including ferroptosis, autophagy, and apoptosis through inducing oxidative stress in human OS cells. The crosstalk and interrelationship between different cell death pathways induced by PEITC in human OS cells are still elusive and needed to be further investigated.

## Data Availability

The relevant data used to support all the findings of this study are included within the article. The primary data of this study may be released upon request to the corresponding author, Prof. P Shang (shangpeng@nwpu.edu.cn, Research & Development Institute of Northwestern Polytechnical University in Shenzhen) and the first author, Dr. H Lv (lvhh2017@nwpu.edu.cn, School of Life Sciences, Northwestern Polytechnical University).

## References

[1] Picci P. (2007). Osteosarcoma (osteogenic sarcoma). *Orphanet Journal of Rare Diseases*.

[2] Luetke A., Meyers P. A., Lewis I., Juergens H. (2014). Osteosarcoma treatment-where do we stand? A state of the art review. *Cancer Treatment Reviews*.

[3] Harrison D. J., Geller D. S., Gill J. D., Lewis V. O., Gorlick R. (2018). Current and future therapeutic approaches for osteosarcoma. *Expert Review of Anticancer Therapy*.

[4] Saraf A. J., Fenger J. M., Roberts R. D. (2018). Osteosarcoma: accelerating progress makes for a hopeful future. *Frontiers in Oncology*.

[5] Rickel K., Fang F., Tao J. (2017). Molecular genetics of osteosarcoma. *Bone*.

[6] Isakoff M. S., Bielack S. S., Meltzer P., Gorlick R. (2015). Osteosarcoma: current treatment and a collaborative pathway to success. *Journal of Clinical Oncology*.

[7] Gill J., Ahluwalia M. K., Geller D., Gorlick R. (2013). New targets and approaches in osteosarcoma. *Pharmacology Therapeutics*.

[8] Manchali S., Chidambara Murthy K. N., Patil B. S. (2012). Crucial facts about health benefits of popular cruciferous vegetables. *Journal of Functional Foods*.

[9] Liu X., Lv K. (2013). Cruciferous vegetables intake is inversely associated with risk of breast cancer: a meta-analysis. *Breast*.

[10] Mitsiogianni M., Amery T., Franco R., Zoumpourlis V., Pappa A., Panayiotidis M. I. (2018). From chemo-prevention to epigenetic regulation: the role of isothiocyanates in skin cancer prevention. *Pharmacology Therapeutics*.

[11] Herr I., Buchler M. W. (2010). Dietary constituents of broccoli and other cruciferous vegetables: implications for prevention and therapy of cancer. *Cancer Treatment Reviews*.

[12] Higdon J. V., Delage B., Williams D. E., Dashwood R. H. (2007). Cruciferous vegetables and human cancer risk: epidemiologic evidence and mechanistic basis. *Pharmacological Research*.

[13] Yuan J. M., Stepanov I., Murphy S. E. (2016). Clinical trial of 2-phenethyl isothiocyanate as an inhibitor of metabolic activation of a tobacco-specific lung carcinogen in cigarette smokers. *Cancer Prevention Research*.

[14] Palliyaguru D. L., Yuan J. M., Kensler T. W., Fahey J. W. (2018). Isothiocyanates: translating the power of plants to people. *Molecular Nutrition & Food Research*.

[15] Ji Y., Kuo Y., Morris M. E. (2005). Pharmacokinetics of dietary phenethyl isothiocyanate in rats. *Pharmaceutical Research*.

[16] Gupta P., Wright S. E., Kim S. H., Srivastava S. K. (2014). Phenethyl isothiocyanate: a comprehensive review of anti-cancer mechanisms. *Biochimica et Biophysica Acta*.

[17] Ioannides C., Konsue N. (2015). A principal mechanism for the cancer chemopreventive activity of phenethyl isothiocyanate is modulation of carcinogen metabolism. *Drug Metabolism Reviews*.

[18] Gupta P., Kim B., Kim S. H., Srivastava S. K. (2014). Molecular targets of isothiocyanates in cancer: recent advances. *Molecular Nutrition & Food Research*.

[19] De Flora S., Ganchev G., Iltcheva M. (2016). Pharmacological modulation of lung carcinogenesis in smokers: preclinical and clinical evidence. *Trends in Pharmacological Sciences*.

[20] Conaway C. C., Wang C. X., Pittman B. (2005). Phenethyl isothiocyanate and sulforaphane and their N-acetylcysteine conjugates inhibit malignant progression of lung adenomas induced by tobacco carcinogens in A/J mice. *Cancer Research*.

[21] Yeh Y. T., Yeh H., Su S. H. (2014). Phenethyl isothiocyanate induces DNA damage-associated G2/M arrest and subsequent apoptosis in oral cancer cells with varying p53 mutations. *Free Radical Biology & Medicine*.

[22] Stan S. D., Singh S. V., Whitcomb D. C., Brand R. E. (2014). Phenethyl isothiocyanate inhibits proliferation and induces apoptosis in pancreatic cancer cells in vitro and in a MIAPaca2 xenograft animal model. *Nutrition and Cancer*.

[23] Satyan K. S., Swamy N., Dizon D. S., Singh R., Granai C. O., Brard L. (2006). Phenethyl isothiocyanate (PEITC) inhibits growth of ovarian cancer cells by inducing apoptosis: role of caspase and MAPK activation. *Gynecologic Oncology*.

[24] Kasukabe T., Honma Y., Okabe-Kado J., Higuchi Y., Kato N., Kumakura S. (2016). Combined treatment with cotylenin A and phenethyl isothiocyanate induces strong antitumor activity mainly through the induction of ferroptotic cell death in human pancreatic cancer cells. *Oncology Reports*.

[25] Jakubikova J., Cervi D., Ooi M. (2011). Anti-tumor activity and signaling events triggered by the isothiocyanates, sulforaphane and phenethyl isothiocyanate, in multiple myeloma. *Haematologica*.

[26] Cheung K. L., Khor T. O., Yu S., Kong A. N. (2008). PEITC induces G1 cell cycle arrest on HT-29 cells through the activation of p38 MAPK signaling pathway. *The AAPS Journal*.

[27] Dixon S. J., Lemberg K. M., Lamprecht M. R. (2012). Ferroptosis: an iron-dependent form of nonapoptotic cell death. *Cell*.

[28] Hatem E., El Banna N., Huang M. E. (2017). Multifaceted roles of glutathione and glutathione-based systems in carcinogenesis and anticancer drug resistance. *Antioxidants & Redox Signaling*.

[29] Lalaoui N., Lindqvist L. M., Sandow J. J., Ekert P. G. (2015). The molecular relationships between apoptosis, autophagy and necroptosis. *Seminars in Cell & Developmental Biology*.

[30] Gao M., Monian P., Pan Q., Zhang W., Xiang J., Jiang X. (2016). Ferroptosis is an autophagic cell death process. *Cell Research*.

[31] Kang R., Tang D. (2017). Autophagy and ferroptosis—what's the connection?. *Current Pathobiology Reports*.

[32] Stockwell B. R., Friedmann Angeli J. P., Bayir H. (2017). Ferroptosis: a regulated cell death nexus linking metabolism, redox biology, and disease. *Cell*.

[33] Gao M., Yi J., Zhu J. (2019). Role of mitochondria in ferroptosis. *Molecular Cell*.

[34] Circu M. L., Aw T. Y. (2010). Reactive oxygen species, cellular redox systems, and apoptosis. *Free Radical Biology & Medicine*.

[35] Moloney J. N., Cotter T. G. (2017). ROS signalling in the biology of cancer. *Seminars in Cell & Developmental Biology*.

[36] Panieri E., Santoro M. M. (2016). ROS homeostasis and metabolism: a dangerous liason in cancer cells. *Cell Death & Disease*.

[37] Hong Y. H., Uddin M. H., Jo U. (2015). ROS accumulation by PEITC selectively kills ovarian cancer cells via UPR-mediated apoptosis. *Frontiers in Oncology*.

[38] Gong A., He M., Krishna Vanaja D., Yin P., Karnes R. J., Young C. Y. (2009). Phenethyl isothiocyanate inhibits STAT3 activation in prostate cancer cells. *Molecular Nutrition & Food Research*.

[39] Bogdan A. R., Miyazawa M., Hashimoto K., Tsuji Y. (2016). Regulators of iron homeostasis: new players in metabolism, cell death, and disease. *Trends in Biochemical Sciences*.

[40] Crielaard B. J., Lammers T., Rivella S. (2017). Targeting iron metabolism in drug discovery and delivery. *Nature Reviews Drug Discovery*.

[41] Kuhn L. C. (2015). Iron regulatory proteins and their role in controlling iron metabolism. *Metallomics*.

[42] Dixon S. J., Stockwell B. R. (2014). The role of iron and reactive oxygen species in cell death. *Nature Chemical Biology*.

[43] Powolny A. A., Bommareddy A., Hahm E. R. (2011). Chemopreventative potential of the cruciferous vegetable constituent phenethyl isothiocyanate in a mouse model of prostate cancer. *Journal of the National Cancer Institute*.

[44] Fan Q., Zhan X., Xiao Z., Liu C. (2015). Phenethyl isothiocyanate enhances adriamycin-induced apoptosis in osteosarcoma cells. *Molecular Medicine Reports*.

[45] Wu W. J., Zhang Y., Zeng Z. L. (2013). *β*-Phenylethyl isothiocyanate reverses platinum resistance by a GSH-dependent mechanism in cancer cells with epithelial-mesenchymal transition phenotype. *Biochemical Pharmacology*.

